# Primate-conserved carbonic anhydrase IV and murine-restricted LY6C1 enable blood-brain barrier crossing by engineered viral vectors

**DOI:** 10.1126/sciadv.adg6618

**Published:** 2023-04-19

**Authors:** Timothy F. Shay, Erin E. Sullivan, Xiaozhe Ding, Xinhong Chen, Sripriya Ravindra Kumar, David Goertsen, David Brown, Anaya Crosby, Jost Vielmetter, Máté Borsos, Damien A. Wolfe, Annie W. Lam, Viviana Gradinaru

**Affiliations:** ^1^ Division of Biology and Biological Engineering, California Institute of Technology, Pasadena, CA, USA.; ^2^ California State Polytechnic University, Pomona, Pomona, CA, USA.

## Abstract

The blood-brain barrier (BBB) presents a major challenge for delivering large molecules to study and treat the central nervous system. This is due in part to the scarcity of targets known to mediate BBB crossing. To identify novel targets, we leverage a panel of adeno-associated viruses (AAVs) previously identified through mechanism-agnostic directed evolution for improved BBB transcytosis. Screening potential cognate receptors for enhanced BBB crossing, we identify two targets: murine-restricted LY6C1 and widely conserved carbonic anhydrase IV (CA-IV). We apply AlphaFold-based in silico methods to generate capsid-receptor binding models to predict the affinity of AAVs for these identified receptors. Demonstrating how these tools can unlock target-focused engineering strategies, we create an enhanced LY6C1-binding vector, AAV-PHP.eC, that, unlike our prior PHP.eB, also works in *Ly6a*-deficient mouse strains such as BALB/cJ. Combined with structural insights from computational modeling, the identification of primate-conserved CA-IV enables the design of more specific and potent human brain–penetrant chemicals and biologicals, including gene delivery vectors.

## Teaser

A conserved receptor is identified as a target for engineered vectors to deliver therapeutics to the brain from the bloodstream.

## INTRODUCTION

The blood-brain barrier (BBB) presents a fundamental bottleneck for the delivery of effective research tools and therapeutics to the central nervous system (CNS) (*1*–*3*). This complex structure, composed mainly of brain endothelial cells along with pericytes, astrocytes, and microglia (*2*, *4*), prevents the passage of large molecules, including gene delivery vectors, such as adeno-associated viruses (AAVs). Some can cross by receptor-mediated transcytosis (*5*, *6*); the rest must be delivered via invasive intracranial injections or technically challenging focused ultrasound (*7*). The rational design of BBB-crossing large molecules has long been hampered by our imperfect understanding of the mechanisms involved in transcytosis, with only a handful of targets, such as transferrin receptor (*8*–*11*), validated for research and therapies (*6*, *12*–*14*).

Directed evolution is a powerful method for generating biomolecules with enhanced fitness for desired properties even with incomplete understanding of the underlying biological systems (*15*). The outcomes of directed evolution libraries can in turn be used to unlock previously unknown biology by probing the mechanism of action of molecules with evolved properties. We decided to apply this paradigm of reverse-engineering directed evolution hits to the accumulating wealth of AAV libraries selected for CNS enrichment after systemic administration (*16*–*25*).

One such improved rodent BBB–crossing AAV capsid is PHP.eB (*26*), which we previously identified by applying Cre recombination–based AAV targeted evolution (CREATE) (*17*) to the AAV9 parent capsid (*27*, *28*). Following systemic injection in genetically divergent mouse strains, PHP.eB can show either potent CNS tropism (as in C57BL/6J, FVB/NCrl, and DBA/2J) or behavior akin to AAV9 (as in BALB/cJ) (*29*–*31*). This is explained by the receptor for PHP.eB, the glycosylphosphatidylinositol (GPI)–anchored protein LY6A, being strongly expressed in C57BL/6J (the directed evolution selection host), FVB/NCrl, and other mouse strains but nonfunctional in the BALB/cJ strain and other mammals (*32*–*34*). Consistently, in nonhuman primates (NHPs) that lack functional LY6A, PHP.eB and related capsids display limited, AAV9-like CNS infectivity (*29*, *35*–*37*). As AAVs have become the vector of choice for human gene therapies, including for the CNS (*38*–*40*), complications from directly applying mouse-evolved AAVs in diverse genetic backgrounds has contributed to a shift toward performing AAV directed evolution in NHPs. In so doing, researchers hope to increase the likelihood of identifying AAVs whose enhanced tropism will translate to humans.

As both preclinical validation and, increasingly, selection of engineered AAV capsids occur in NHPs (*37*, *41*, *42*), problems arise. The animals’ scarcity (*43*, *44*) and cost slow the identification of promising capsids, and the risk of species-specific AAVs (that behave differently in NHPs and humans) entering clinical trials remains. Nevertheless, examples are beginning to accumulate not only of capsids that can cross both rodent and NHP BBB (*37*) but also, importantly, of ones that can cross the macaque but not the rodent BBB (*42*). Collectively, this diverse set of capsids engineered over the past decade by many groups represents, through their yet-unexplored mechanisms of action, an unprecedented opportunity to start discovering targets for crossing the BBB across strains and species.

Here, we demonstrate a path forward to identifying BBB-crossing mechanisms by understanding how brain-tropic AAVs, such as those previously identified by directed evolution in mice (*20*, *22*, *24*, *37*), reach their destination from the bloodstream. Focusing on AAVs whose enhanced CNS infectivity upon systemic injection in mice is conserved across strains (*20*, *22*, *24*), we identified two receptors for potent BBB crossing by engineered AAVs: murine-restricted LY6C1 and primate-conserved carbonic anhydrase IV (CA-IV). As a proof of concept for receptor-informed directed evolution, we targeted LY6C1 to generate AAV-PHP.eC, a mechanistically distinct BBB-crossing AAV for murine models lacking functional LY6A (e.g., BALB/cJ). To further unlock target-driven vector engineering strategies, we used a recently developed computational approach (*45*) that uses AlphaFold-Multimer (*46*, *47*) to screen peptides against potential receptors in silico and generated interaction models of AAVs and their receptors that are challenging to solve experimentally and that may inform human BBB-crossing drug development.

## RESULTS

### Identification of engineered AAVs that do not use LY6A

**Fig. 1.**
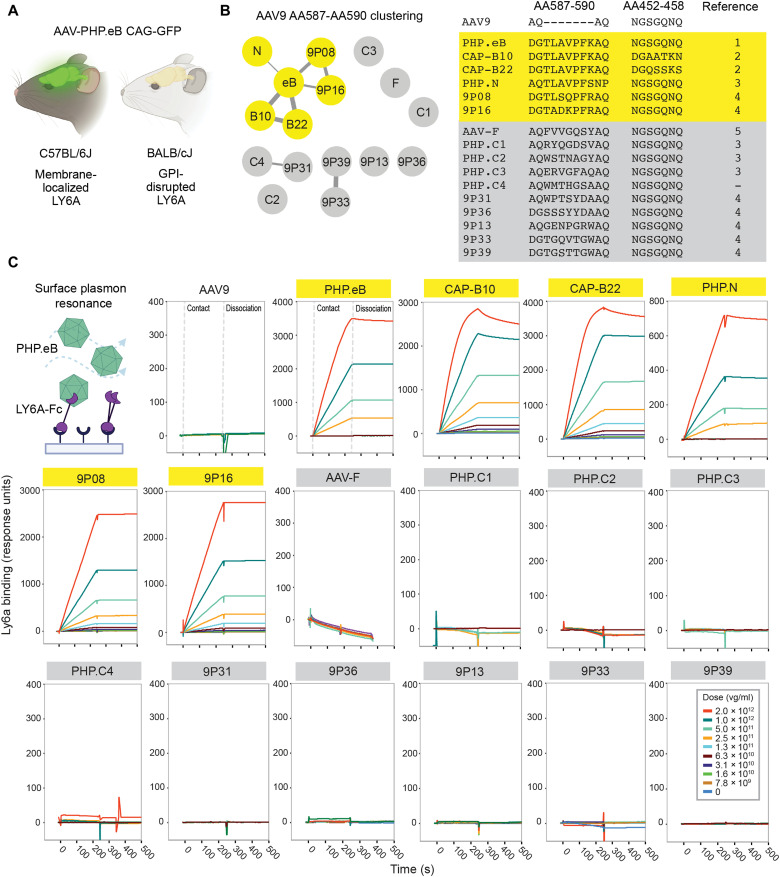
Identifying engineered AAVs that do not use LY6A for BBB crossing. (**A**) AAV-PHP.eB can efficiently cross the BBB using membrane-localized LY6A in C57BL/6J mice but not GPI-disrupted LY6A in BALB/cJ mice. (**B**) Clustering analysis of CNS-tropic engineered AAV insertion sequences. Thickness of connections represents degree of relatedness between nodes. AAVs in yellow show a high degree of relatedness to PHP.eB. AAVs in gray show little relation to PHP.eB or each other. The peptide sequences in two engineered regions (AA587 to AA590 and AA452 to AA458 of the AAV9 capsid for each variant are shown. References: 1: Chan *et al.* (*26*), 2: Goertsen *et al.* (*37*), 3: Ravindra Kumar *et al.* (*22*), 4: Nonnenmacher *et al.* (*24*), and 5: Hanlon *et al.* (*20*). (**C**) Surface plasmon resonance (SPR) of engineered AAVs binding to LY6A-Fc captured on a protein A chip. Data are representative of two independent experiments. v.g., viral genome.

LY6A is the receptor responsible for the enhanced CNS tropism of PHP.B and PHP.eB in mice (*32*–*34*), and one strain-specific single-nucleotide polymorphism (SNP) disrupts its GPI-anchored membrane localization (Fig. 1A) (*34*). Previously, we applied our M-CREATE directed evolution selection platform to a library of AAV9 variants (with seven-amino-acid insertions at position 588 of capsid variable region VIII) and identified a family of engineered AAVs with diverse CNS tropisms and a shared sequence motif, typified by their founding member, AAV-PHP.B (Fig. 1B) (*22*). The variants’ enhanced CNS potency is lacking in BALB/cJ mice, a phenomenon explained by their shared reliance on LY6A for BBB crossing (*32*–*34*). Recently, multiple engineered AAVs outside the PHP.B sequence family which retain their enhanced CNS tropism in BALB/cJ mice were identified (*20*, *22*, *24*). Before attempting to find cognate receptors for these AAVs, we first sought to confirm their independence of LY6A.

To directly probe potential LY6A binding interactions, we performed surface plasmon resonance (SPR). To ensure the detection of even weak interactions, we dimerized LY6A by fusion to Fc, immobilized it at high density on a protein A chip, and tested each AAV analyte across a range of concentrations (Fig. 1C). As expected, AAV9 showed no evidence of binding at any concentration, and PHP.B sequence family members all showed strong binding interactions with LY6A. While precise affinities could not be determined because of the effects of avidity and mass transport, the interaction profiles were consistent with subnanomolar affinities. Conversely, all BALB/cJ-enhanced engineered AAVs were indistinguishable from AAV9, exhibiting no detectable interaction with LY6A.

### Construction of a cell culture screen for putative receptors of engineered AAVs

**Fig. 2.**
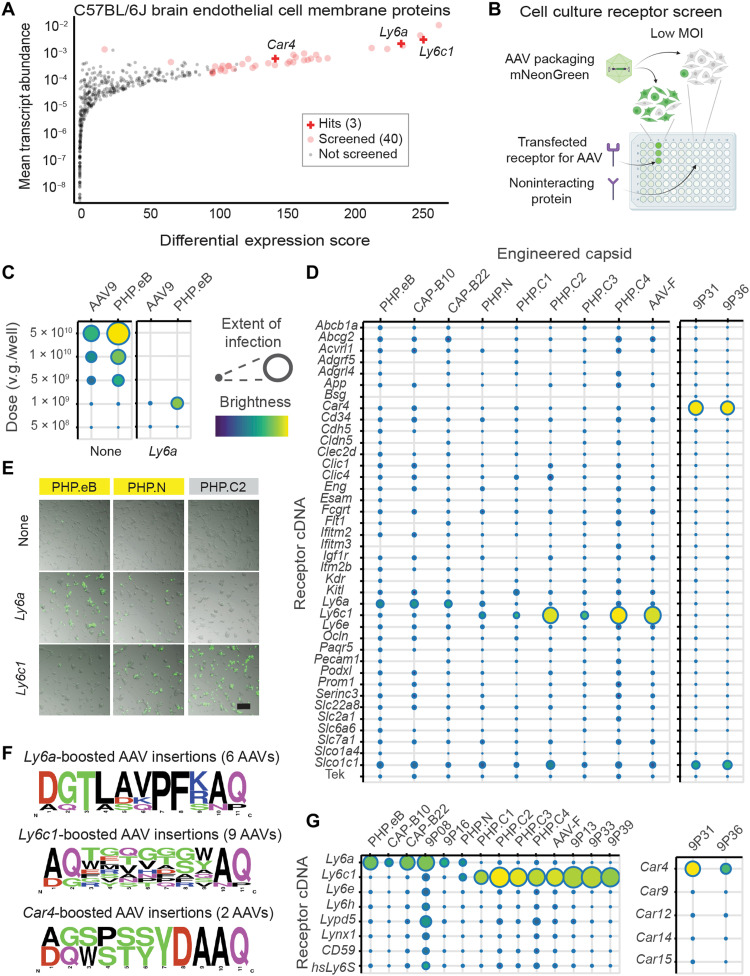
Previously unidentified receptors enhance infectivity of engineered AAVs in vitro. (**A**) Plot of membrane protein differential expression score in endothelial cells versus mean transcript abundance in endothelial cells constructed from single-cell RNA sequencing of C57BL/6J cortex. Proteins selected for cell culture screening (table S1) are indicated by red circles, and three hits are marked with red crosses. (**B**) Potential receptors were screened in cell culture by comparing AAV fluorescent protein transgene levels at low multiplicity of infection (MOI) in cells transfected with the cDNA for each potential receptor and in mock-transfected cells. (**C**) Dose dependence of AAV9 and PHP.eB packaging CAG-mNeonGreen in HEK293T cells in 96-well plates. At 1 × 10^9^ v.g. per well, PHP.eB shows *Ly6a*-enhanced infection. Scales show extent of infection (max, 0.75; min, 0.03) and total brightness per signal area (max, 0.79; min, 0.39). (**D**) Potency of engineered AAVs for HEK293T cells transfected with the potential receptor cDNA panel. Extent of infection (left: max, 0.33; min, 0.001; right: max, 0.55; min, 0.03) and total brightness per signal area (left: max, 0.55; min, 0.06; right: max, 0.61: min, 0.11). (**E**) Representative images of HEK cells cultured in 96-well plates, either mock-transfected (None) or transfected with a potential receptor, infected with 1 × 10^9^ v.g. per well (yellow label background indicates LY6A binding) packaging CAG-mNeonGreen and imaged 24 hours after transduction. An overlay of bright-field and fluorescence images is presented. Scale bar, 200 μm. (**F**) Amino acid frequencies by position among engineered AAVs found to have infectivity enhancements with *Ly6a* (six viruses), *Ly6c1* (nine viruses), and *Car4* (two viruses). (**G**) Potency of engineered AAVs for HEK293T cells transfected with LY6 and CAR family potential receptors. Extent of infection (left: max, 0.51; min, 0.03; right: max, 0.52; min, 0.05) and total brightness per signal area (left: max, 0.75; min, 0.13; right: max, 0.79; min, 0.34).

Reasoning that a receptor that engineered AAVs can co-opt for efficient BBB crossing is likely to be both highly expressed and highly specific to the endothelial cells of the brain, we analyzed previously collected single-cell RNA sequencing data from dissociated C57BL/6J brain tissue (Fig. 2A) (*48*). We investigated gene expression levels within CNS endothelial cell clusters and differential expression compared to most other CNS cell type clusters. Genes were filtered to select only those annotated in UniProt as localized to the plasma membrane before calculating their endothelial cell differential expression score in Scanpy. This score was then plotted against each transcript’s mean abundance, revealing a long tail of highly expressed and highly specific CNS endothelial membrane proteins. Encouragingly, *Ly6a* appeared at the far end of this tail. From this analysis, we selected a panel of 40 abundant and specific candidate receptors (table S1).

We and others have observed that expression of membrane-localized LY6A in human embryonic kidney (HEK) 293 cells selectively improves the potency of PHP.eB infection compared to AAV9 at low multiplicity of infection (MOI), with extent of infection and brightness of infected cells markedly increased (fig. S1A) (*34*). Hypothesizing that this property is likely to be conserved among BBB-crossing receptors, we made this behavior the basis of a receptor transient overexpression screen (Fig. 2, B and C). We cloned the C57BL/6J coding sequence of each of the 40 candidate receptors into a mammalian expression plasmid and tested each against engineered AAVs in triplicate at two different doses (Fig. 2, A and D). PHP.eB and *Ly6a* served as a positive control.

As expected, we found that all members of the PHP.B sequence family showed a marked boost in infectivity in *Ly6a*-transfected cells compared to untransfected cells, while *Ly6a*-independent AAVs performed identically in both conditions (Fig. 2, D and E, and fig. S1B). The infectivity of all *Ly6a*-dependent capsids was boosted to a similar extent (fig. S2B).

### Identification of LY6C1 and CA-IV as receptors for BBB crossing of *Ly6a*-independent engineered AAVs

We observed boosts in infectivity for all tested *Ly6a*-independent AAVs with previously unidentified receptors (Fig. 2D). Unexpectedly, given their different sequence families, all of the initial *Ly6a*-independent AAVs responded to the same candidate receptor, *Ly6c1* (Fig. 2, D to G, and fig. S1B). In addition, despite its *Ly6a*-dependent pattern of CNS infectivity across murine strains (*22*), PHP.N also exhibited enhanced infectivity in *Ly6c1*-transfected cells. At a higher dose, PHP.N outperformed both AAV9 and PHP.eB in CBA/J mice (fig. S3), which express a GPI-disrupted LY6A. While polymorphisms in *Ly6c1* exist between mouse strains [as shown by a published analysis of the 36 mouse strains for which whole genomes are available (*34*)], we found that, unlike *Ly6a*, none of the polymorphisms were predicted to disrupt GPI anchoring of LY6C1 to the plasma membrane using PredGPI (*49*). To determine the specificity of the LY6C1 interaction, we performed a follow-up screen with additional closely related and CNS-expressed LY6 superfamily members and found no cross-reactivity of *Ly6c1*-dependent AAVs with other LY6 proteins (Fig. 2G). CAP-B10 and CAP-B22, which have seven amino acid substitutions in capsid variable region IV and show enhanced potency in adult marmosets (*37*), did not exhibit any additional receptor interactions that would explain their NHP tropism in either of the screens described above or a third screen with marmoset CNS-expressed LY6 family members (fig. S2C) nor did any LY6-interacting AAV cross-react with the recently described human LY6S, a close relative of murine LY6A (Fig. 2G) (*50*). While we used validated candidate receptor clones (table S1), it is possible that lack of membrane expression could result in a false negative in our screens.

**Fig. 3.**
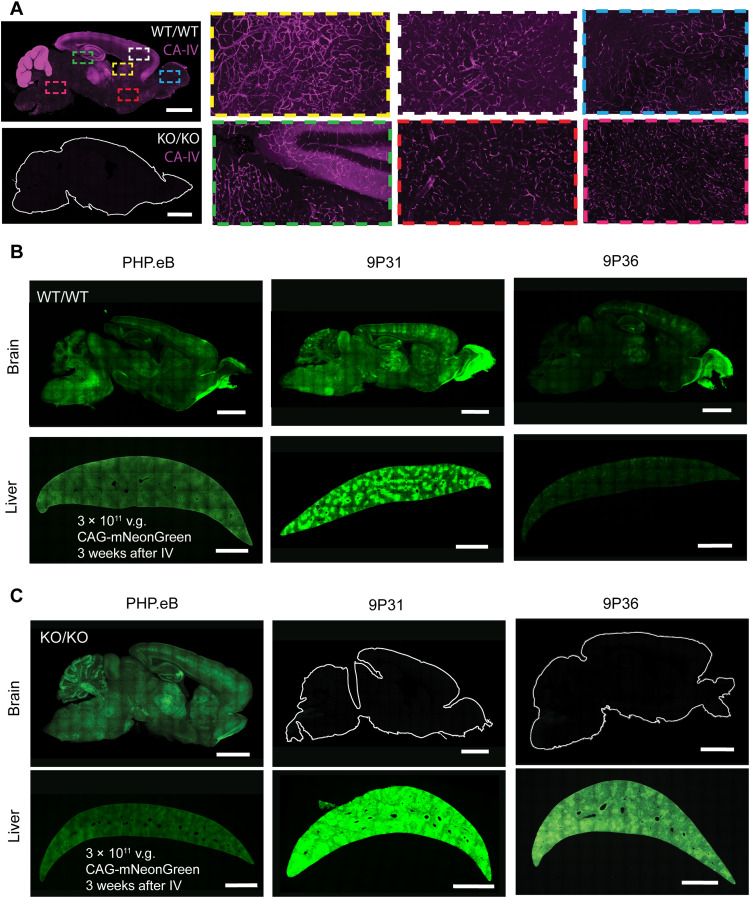
CA-IV is required for the CNS potency of *Car4*-dependent AAV. (**A**) Immunostaining for CA-IV in the brains of WT/WT and KO/KO *Car4* mice. Magnified regions from WT/WT demonstrate endothelial expression across diverse brain regions. (**B**) AAV-PHP.eB, 9P31, and 9P36 packaging mNeonGreen under the control of the ubiquitous CAG promoter were intravenously administered to WT/WT *Car4* mice at a dose of 3 × 10^11^ v.g. per animal (*n* = 3 per condition). Three weeks after administration, transgene expression was assayed by mNeonGreen fluorescence throughout the brain and liver. (**C**) AAV-PHP.eB, 9P31, and 9P36 packaging mNeonGreen under the control of the ubiquitous CAG promoter were intravenously administered to KO/KO *Car4* mice at a dose of 3 × 10^11^ v.g. per animal (*n* = 3 per condition). Three weeks after administration, transgene expression was assayed by mNeonGreen fluorescence throughout the brain and liver. Scale bars, 2 mm.

We also expanded the LY6 follow-up screen to include a second set of seven previously identified engineered AAVs (9P) (*24*), which yielded an additional five LY6A and LY6C1-interacting AAVs (Fig. 2G). The 9P AAVs 9P31 and 9P36, however, did not display enhanced infectivity with any LY6 protein in the panel and so were tested on the full receptor panel (Fig. 2D). Both 9P31 and 9P36 displayed an infectivity boost with the GPI-linked enzyme CA-IV (encoded by *CA4* in human and *Car4* in mouse) (Fig. 2D and fig. S1B). This interaction was specific to CA-IV among membrane-associated CAs (Fig. 2G). As with *Ly6c1*, polymorphisms in *Car4* across mouse strains are not predicted to affect GPI anchoring to the plasma membrane (*34*, *49*). Unlike mouse-restricted *Ly6a* and *Ly6c1*, *CA4/Car4* is conserved throughout vertebrates, including NHPs and humans (*51*–*53*). Therefore, we chose to confirm our screen results in vivo using *Car4* knockout (KO) mice (B6.129S1-Car4tm1Sly/J, the Jackson Laboratory (JAX), strain no. 008217). Immunofluorescence confirmed that CA-IV is strongly expressed throughout the brain vasculature of homozygous wild-type (WT)/WT mice and completely absent in KO/KO (Fig. 3A). When dosed with 3 × 10^11^ viral genomes (v.g.) per animal, PHP.eB, 9P31, and 9P36 all strongly expressed in both the brain and liver of WT mice (Fig. 3B). Under the same conditions in KO/KO mice, PHP.eB was unaffected, whereas both 9P31 and 9P36 completely lack enhanced CNS tropism (Fig. 3C and fig. S3B). On the other hand, liver tropism, which is governed by AAV9’s naturally evolved receptor interactions, was decoupled from this effect, with all three viruses showing strong transduction in KO/KO mice.

Notably, every AAV we tested showed a moderate boost in infectivity in cells transfected with *Slco1c1* (also known as *Oatp1c1*), an integral membrane anionic transporter (Fig. 2D). Unlike for other potential receptors, however, the mNeonGreen signal was weak and diffuse, extending beyond cell boundaries (fig. S1C), suggesting a transgene export or cell health phenotype. The universality of this effect and the specificity of *Slco1c1* expression in the brain suggest a possible role in the weak BBB transcytosis of parent AAV9. None of the other 36 candidate receptors produced a meaningful infectivity boost for any of the 16 engineered AAVs screened.

### Directed evolution of an improved *Ly6c1*-dependent capsid

Next, we aimed to demonstrate a proof of concept for receptor-targeted directed evolution. For this purpose, we chose the murine receptor LY6C1 (versus the human CA-IV) due to prompt availability of (i) strains with clear BBB differences, e.g. LY6A, and (ii) diverse Cre-transgenic animals for M-CREATE selections that, for now, are not available for CA-IV in other species. In addition, given the mixed backgrounds of preclinical animal models, it remains important to have mechanistically distinct gene delivery vectors for rodents. We therefore sought to engineer a single optimized capsid for broad adoption as a research tool in GPI-disrupted LY6A mouse strains, a still unmet need, using the M-CREATE method we previously developed for AAV capsid directed evolution (*22*). This method uses Cre-dependent AAV genome recovery from desired tissues and cell types after in vivo selection in Cre-transgenic mice and allows deep characterization of selected capsids across various cell types and tissues.

**Fig. 4.**
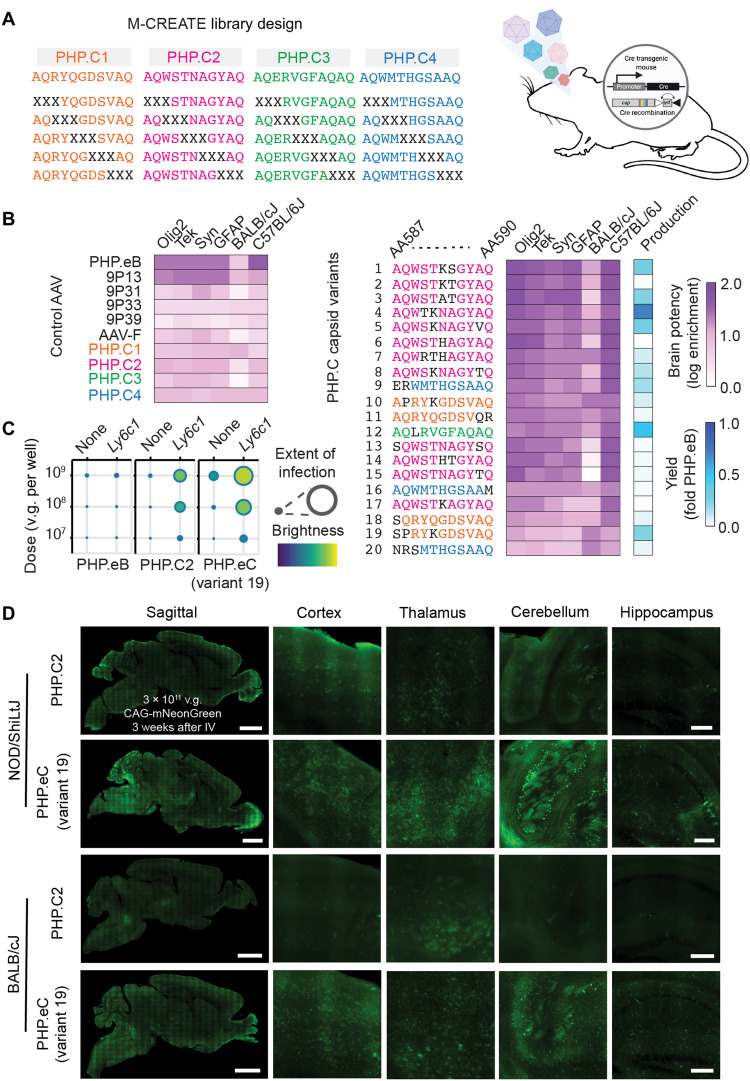
Engineering an enhanced *Ly6c1*-dependent AAV. (**A**) AAV library design strategy. Scanning 3-amino-acid libraries of PHP.C1 to C4 were constructed as shown with Xs indicating the position of NNK codons. AAVs were pooled, and two rounds of M-CREATE selections were performed in 6- to 8-week-old Cre-transgenic mice. (**B**) Round 2 selection brain enrichments for diverse Cre-transgenic and WT mice of selected control AAVs and 20 capsid variants selected for further study. Yield was determined during small-scale production for final screening across strains. (**C**) Potency in cell culture infectivity assay of PHP.eB, PHP.C2, and PHP.eC (variant 19) in HEK293T cells transfected with mock (none) or *Ly6c1* receptor cDNA, demonstrating that PHP.eC retains LY6C1 interaction. Extent of infection (max, 0.93; min, 0.02). Total brightness per signal area (max, 0.74; min, 0.16). (**D**) PHP.C2 and PHP.eC packaging mNeonGreen under the control of the ubiquitous CAG promoter were intravenously administered to nonobese diabetic (NOD)/ShiLtJ and BALB/cJ mice at a dose of 3 × 10^11^ v.g. per animal (*n* = 3 per condition). Three weeks after administration, transgene expression was assayed by mNeonGreen fluorescence throughout the brain and liver, demonstrating PHP.eC’s increased potency. Scale bars, 2 mm (sagittal image) and 250 μm (brain region).

Using M-CREATE, we constructed scanning 3-amino-acid substitution capsid libraries in the chemically diverse LY6C1-interacting variants PHP.C1, PHP.C2, PHP.C3, and PHP.C4 and pooled these libraries for two rounds of selection in Syn-Cre mice (Fig. 4A). In the second round of selection, we also included Olig2-Cre, Tek-Cre, and glial fibrillary acidic protein (GFAP)–Cre mice, as well as WT C57BL/6J and BALB/cJ mice, so that we could detect potential differences in enhancement between these strains or cell types during selection among the variants (Fig. 4B). While PHP.C2 variants dominated both rounds of selection in the C57BL/6J background (Cre-dependent or not), PHP.C1 variants dominated the round 2 Cre-independent selection in BALB/cJ (fig. S4). Following selection, we individually produced and characterized top-performing variants. AAV-PHP.eC (variant 19), evolved from PHP.C1, retained LY6C1 interaction in cell culture (Fig. 4C) and outperformed PHP.C2 in multiple mouse strains with membrane-disrupted LY6A (Fig. 4D). Immunohistochemistry confirmed that PHP.eC strongly infects neurons and astrocytes across strains (fig. S5A). PHP.eC thus provides a potent tool for transgene delivery in mouse strains without membrane-localized LY6A.

### AlphaFold-based methods to identify receptor binding peptides and engineered AAV binding poses in silico

**Fig. 5.**
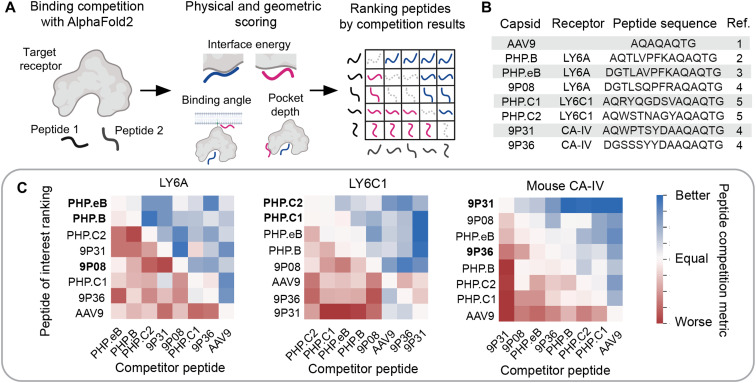
In silico ranking of AAV variants by their receptor binding propensities. (**A**) Overview of AlphaFold-based in silico Automated Pairwise Peptide-Receptor Analysis for Screening Engineered AAVs (APPRAISE-AAV for short) (*45*). Surface peptides from AAV variants are put in pairwise binding competition using AlphaFold-Multimer. A peptide competition metric is calculated according to each peptide’s interface energy, binding angle, and pocket depth (see Materials and Methods for details) before being assembled into broader ranked matrices of interaction likelihood. Competition results reflect the relative peptide binding probability encoded in the AlphaFold neural network. (**B**) Table of engineered AAV capsids, their confirmed receptor, and the capsid peptide sequence used in APPRAISE-AAV. References: 1: Gao *et al.* (*27*), 2: Deverman *et al.* (*17*), 3: Chan *et al.* (*26*), 4: Nonnenmacher *et al.* (*24*), and 5: Ravindra Kumar *et al.* (*22*). (**C**) Matrices ranking AAV peptides by their average competition metric over 10 replicate conditions for LY6A, LY6C1, and mouse CA-IV. AAV peptide labels in bold indicate those experimentally identified to interact with the corresponding receptor. Metric values out of range (−100 to 100) were capped to range limits.

Having identified a panel of receptor and AAV capsid pairings, we aimed to see whether we could capitalize on rapid advances in protein structure prediction to generate binding poses for engineered AAVs and their cognate receptors. We began by applying an AlphaFold-based computational method (*46*, *47*) for Automated Pairwise Peptide Receptor Analysis for Screening Engineered AAVs (APPRAISE-AAV for short) (*45*). Inspired by recent work (*54*, *55*), this method uses AlphaFold-Multimer to place surface-exposed peptides spanning mutagenic insertions (AA587 to AA594) from two distinct AAV variants in competition to interact with a potential receptor (Fig. 5A). This comprises the minimal peptide that encompasses the solvent-exposed residues of capsid variable region VIII. A combination of physical and geometric scoring parameters that include interface energy, binding angle, and binding pocket depth calculations are used to generate a peptide competition metric. Results from these individual pairwise competitions can be assembled into larger matrices that rank sets of AAV capsid insertion peptides according to their receptor binding probability encoded in the AlphaFold neural network. When applied to the receptors identified in our screens, we found that the experimentally verified LY6A, LY6C1, and CA-IV targeting peptides rise to the top of their respective rankings (Fig. 5, B and C). Some false negatives were also observed, however, as in 9P08 with LY6A or 9P36 with CA-IV.

In addition to predictions of whether a peptide binds to a receptor, we can also computationally interrogate the structural details of the binding interaction. We generated binding poses by pairing the top AAV insertion peptide with its receptor and validated the binding pose for each pairing by repeating our cell culture screen with receptors containing point mutations hypothesized to disrupt the high-confidence region of the binding interface [as determined by the per-residue estimated model confidence–predicted local distance difference test (pLDDT) score and consistency between replicate models] in these predicted poses.

**Fig. 6.**
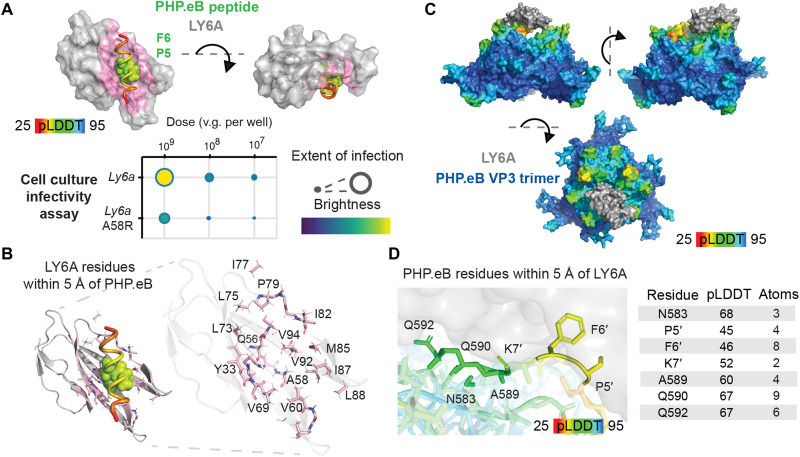
Engineered AAV interactions with LY6A. (**A**) AlphaFold-Multimer–predicted LY6A-PHP.eB peptide complex structure. PHP.eB peptide is color-coded by predicted local distance difference test (pLDDT) score, a per-residue estimate of the model confidence. The highest confidence side chains, P5′ and F6′, are shown as spheres. LY6A A58R mutation, chosen to disrupt the predicted peptide interaction, resulted in reduced potency in the cell culture infectivity assay. Extent of infection (max, 0.29; min, 0.03) and total brightness per signal area (max, 0.61; min, 0.16). (**B**) LY6A residues with at least two atoms within 5 Å of the modeled PHP.eB peptide. (**C**) Complete model of the PHP.eB trimer and LY6A complex. The AlphaFold-Multimer structural prediction from (A) was combined with a capsid monomer-receptor structural prediction and optimized using Rosetta Remodel within the context of the AAV trimer (fig. S6A). (**D**) Zoomed-in view of the PHP.eB-LY6A binding interface in modeled PHP.eB-LY6A complex and PHP.eB residues with at least two atoms within 5 Å of LY6A.

We began by modeling the interaction of PHP.eB with LY6A so that the wealth of existing experimental data on this interaction (*56*–*58*) could be used to build confidence in these methods before their application to LY6C1 and CA-IV receptor interactions. The PHP.eB peptide is predicted to nestle in a groove in LY6A, forming strong interactions at Pro5′ and Phe6′ (Fig. 6A) with several LY6A residues (Fig. 6B). We therefore introduced a point mutation in this groove, LY6A Ala58Arg, without affecting LY6A membrane localization or antibody epitope, and found that it disrupts PHP.eB’s enhanced infectivity with the WT receptor (fig. S5B). This experimental result further bolsters confidence in APPRAISE-AAV rankings in silico.

To gain a full picture of the AAV-receptor interaction, we next modeled the PHP.eB insertion peptide and LY6A receptor complex within the context of the AAV capsid threefold symmetry spike. This structure is challenging for standard modeling tools because of the large size of an AAV capsid (~200 kDa per trimer) as well as the often weak and dynamic binding interactions between engineered capsids and receptors [micromolar affinities possible without avidity (*57*)]. AlphaFold-Multimer failed to capture direct contact between fLY6A and PHP.eB capsid in either monomer or trimer configurations. To address this challenge, we developed an integrative structure modeling pipeline. In this pipeline, an initial model of an AAV capsid trimer predicted using AlphaFold-Multimer is structurally aligned with an AlphaFold-predicted model of the receptor with the AAV peptide through the high-confidence Pro5′ and Phe6′ residues of the peptide insertion and RosettaRemodel (*59*) optimization of the linking peptide residues within the context of the AAV capsid threefold symmetry spike (fig. S6A). This complete binding model (Fig. 6C) provides a snapshot for a dynamic interaction that has thus far proven resistant to high-resolution structural characterization (*57*).

The PHP.eB-LY6A computational model is consistent with available experimental results. RMSD (root mean square deviation) between the computational PHP.eB model and a cryo–electron microscopy (cryo-EM)–based model (*57*) [Protein Data Bank (PDB) ID: 7WQO] is 0.36 Å. RMSD increases in PHP.eB’s engineered loop to 1.36 Å. The only high-confidence deviation from cryo-EM structures of uncomplexed PHP.eB is the side chain of Phe6′, which shows no substantial electron density, indicating flexibility, but forms a stable interaction with LY6A in our model (fig. S6B, right). The high-confidence prediction of Pro5′ and Phe6′ aligns with recent evidence showing that PFK 3-amino-acid insertion alone is sufficient to gain LY6A binding (*58*). While LY6A can bind any insertion loop of a trimer, additional interactions induce steric clashes, supporting a ratio of one LY6A per capsid trimer. A PHP.eB-LY6A complex ensemble image forced to contain 60 bound copies of LY6A resembles a recently reported cryo-EM map, whose analysis pipeline averaged over all 60 singly occupied binding sites to form a composite map (fig. S6C) (*57*). The computational model shows that a single copy of both LY6A and AAV receptor (AAVR) PKD2 domains may bind to the same threefold spike simultaneously without clashing (fig. S6D), in agreement with saturation binding experiments (*57*). Consistent with previous work showing that the LY6A SNP D63G does not affect PHP.eB binding (*34*), the residue is greater than 10 Å from the PHP.eB peptide atoms in the computational model. The PHP.eB-LY6A complex model includes several interactions involving AAV insertion–adjacent residues, which is consistent with a previous report (Fig. 6D) (*56*).

**Fig. 7.**
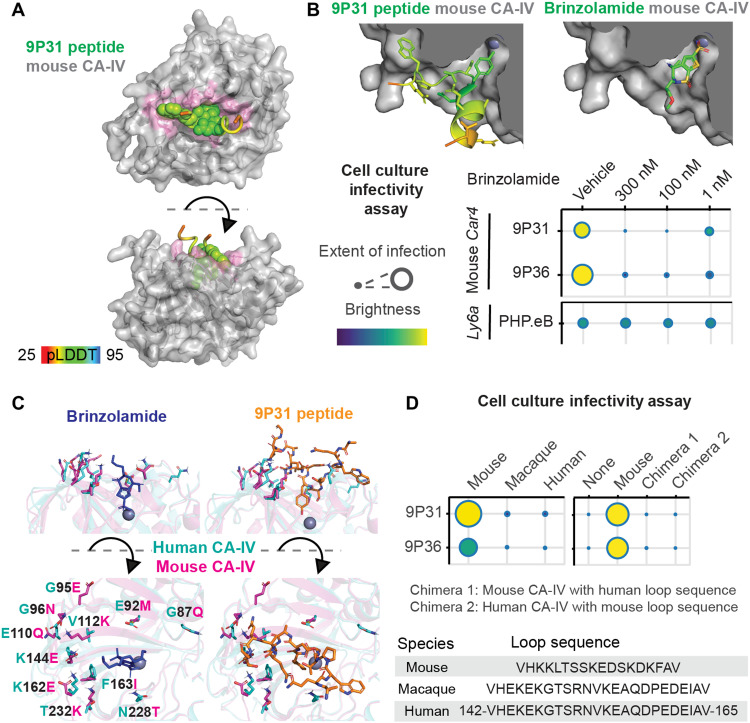
Engineered AAV interactions with CA-IV. (**A**) AlphaFold-Multimer–predicted mouse CA-IV–9P31 peptide complex structure. 9P31 peptide is color-coded by pLDDT score at each residue with the highest confidence side chains shown as spheres. (**B**) Cut-away view of mouse CA-IV catalytic pocket with modeled 9P31 peptide binding pose (top left) and crystallographic brinzolamide binding pose (PDB ID: 3ZNC, top right). Cell culture infectivity assay of brinzolamide’s effects on engineered AAVs (bottom). Extent of infection (max, 0.63; min, 0.04) and total brightness per signal area (max, 0.75; min, 0.18). (**C**) Views of amino acid side chains that differ between mouse (PDB ID: 3ZNC) and human (PDB ID: 1ZNC) CA-IV in relation to brinzolamide and 9P31 peptide binding poses. (**D**) Potency in cell culture infectivity assay of 9P31 and 9P36 in HEK293T cells transfected with mouse, rhesus macaque, or human CA-IV receptors, as well as two chimeric receptors of mouse and human CA-IV that exchange the loop sequences depicted. Extent of infection (left: max, 0.52; min, 0.05; right: max, 0.65; min, 0.03) and total brightness per signal area (left: max, 0.78; min, 0.46; right: max, 0.75; min, 0.13).

Having validated the structural modeling methods against experimental data for the PHP.eB LY6A interaction, we next applied them to the receptors identified in this work. Unlike for LY6A and CA-IV, the predicted binding pose for PHP.C2 peptide with LY6C1 was found to vary with the version of AlphaFold-Multimer used, with v1 predictions closely matching mutational data from our cell culture infectivity assay (fig. S6E). This complementarity between versions has been reported previously (*60*). In mouse CA-IV, 9P31 peptide occupies the catalytic pocket of the enzyme (Fig. 7A). The 9P31 tyrosine residue shared with 9P36 approaches the enzyme active site, and 9P31’s divergent tryptophan is situated in an ancillary pocket (Fig. 7B). This predicted binding pose is competitive with the binding site of brinzolamide (PDB ID: 3NZC) (*61*), a broad CA inhibitor that is prescribed for glaucoma (*62*). In the cell culture infectivity assay, brinzolamide shows a dose-dependent inhibition of 9P31 and 9P36 potency, while PHP.eB is unaffected (Fig. 7B). The smaller brinzolamide binds deep in the catalytic core of CA-IV, where side chains are largely conserved between species (Fig. 7C). 9P31 peptide, however, extends to the surface of the enzyme where there is considerable sequence divergence that prevents cross-reactivity across species. Thus, while brinzolamide binds to both mouse and human CA-IV (*61*, *63*), 9P31 and 9P36 are selective for mouse CA-IV (Fig. 7D). Chimeric receptors that swap a highly divergent loop of the 9P31 binding site show that this region is necessary but not sufficient to control 9P31 and 9P36 potency. Engineering a human CA-IV–binding AAV with optimal BBB-crossing properties is both critically important and not trivial without ready in vivo model systems for validation, as illustrated by the extensive, multi-year efforts realizing transferrin receptor’s potential (*8*, *9*, *64*–*66*).

## DISCUSSION

The BBB restricts access to the CNS by research tools and therapeutics, limiting our ability to study and treat the brain (*1*–*4*). Here, we sought to expand the roster of protein targets through which biologicals and chemicals may access the CNS by finding cognate receptors for engineered AAVs selected through directed evolution for enhanced brain potency. While directed evolution methods have identified several engineered AAVs with enhanced tissue potency after systemic injection (*25*), the mechanisms by which these engineered AAVs gain their enhancements are, with a few recent notable exceptions (*41*, *67*, *68*), largely unknown. The strain dependence and murine restriction of PHP.eB’s LY6A interaction (*32*–*34*) accelerated a push toward NHPs for engineered capsid identification and validation for translational vectors. However, the increasing embrace of engineered AAV capsids for human gene therapy clinical trials (*39*, *40*), coupled with the scarcity and costs of NHPs (*43*, *44*), highlights the need for higher confidence and throughput methods to validate engineered AAVs with diverse, and conserved, mechanisms for crossing the BBB. By screening a curated pool of 40 candidate receptors selected for the intersection of their CNS expression level and endothelial cell specificity, we were able to identify LY6C1 and CA-IV as molecular receptors for enhanced BBB crossing of 10 *Ly6a*-independent engineered AAVs (as well as *Ly6a*-dependent PHP.N). These findings allow for more efficient allocation of NHPs, inform future directed evolution library designs, and enable receptor-guided engineering directly for human protein interaction.

Neither LY6C1 nor CA-IV had been identified as among the most enriched proteins in CNS endothelial cells compared to peripheral endothelial cells (*69*). Given the distinct capsid sites for peptide insertion and galactose (*70*) or AAVR interaction (*71*, *72*) and the simultaneous AAVR PKD2 and LY6A interaction predicted by our model, it is likely that the receptors identified here work in concert with AAV9’s endogenous interaction partners to shape each AAV variant’s tropism.

While differences in their capsid insertion sequences suggested potentially diverse mechanisms for crossing the BBB, nine capsids were found to interact with the same receptor, LY6C1. Differences in potency between AAVs in mouse strains can be dose dependent, as we saw with PHP.N. This suggests that in some cases, a cell culture screen can be sensitive to interactions that might only become functionally relevant in vivo at higher doses. Unlike LY6A, none of the sequence polymorphisms are predicted to interfere with LY6C1 GPI-anchoring and thus membrane localization. LY6C1 expression levels are also consistently high across inbred mouse strains but are significantly lower in recently wild-derived mouse strains (*73*). Together, these features suggest that LY6C1-utilizing AAVs may be useful research tools across genetically diverse mouse strains.

While we confirmed LY6A interaction for CAP.B10 and CAP.B22, no additional interactions were identified. Thus, the mechanism by which these capsids (in contrast to PHP.B/eB) endow enhanced CNS potency in marmoset remains unclear (*37*). It is possible that selecting a variable region IV library in the context of the PHP.eB variable region VIII insertion enriched for alternative receptor interactions outside the most abundant endothelial cell proteins and the LY6 family. If an additional receptor can cooperatively enhance BBB crossing with LY6A, it will enjoy outsized impact on CNS potency compared to acting alone. Thus, selection strategies promoting cooperativity may be used to uncover lower likelihood receptors in mice and promote mechanistic diversity.

That two members of the LY6 protein superfamily predominate as binding partners in CNS selections with AAV9 variable region VIII insertion libraries suggests a special complementarity between this library design and the LY6 protein fold. This notion is supported by recent work suggesting additional interaction sites between WT AAV9 regions of AAV-PHP.eB and LY6A (*56*). Using our integrative modeling pipeline, we generated a complete, experimentally validated receptor complex model for PHP.eB with LY6A, which has otherwise resisted high-resolution structural characterization (*57*). This model illustrates the complementarity of PHP.eB to LY6A and predicts additional interactions outside of the insertion peptide. This insight provides opportunities for improved capsid engineering by both rational design (via in vitro selection for LY6 family members with desirable expression patterns or conservation across species) and directed evolution (via negative selection prescreens against purified LY6 family proteins to encourage other BBB-crossing solutions). The APPRAISE-AAV in silico method is well suited to these screens and can be readily applied to any existing engineered capsid library dataset to mine for capsid variants likely to interact with a chosen target receptor, including CA-IV. Our modeling pipeline also provides high-confidence binding models for receptor complexes with AAVs that have proven difficult to resolve structurally. We note that the APPRAISE methodology is not limited to AAVs (*45*), and the pipeline for generating complexes with full AAV trimer structures may readily be used to guide the translation of engineered peptide insertions identified through directed evolution in AAVs to other protein modalities (*63*).

In addition to the LY6C1-interacting AAVs, our cell culture screen also identified two AAVs, 9P31 and 9P36, with pronounced infectivity boosts with CA-IV. CA-IV, also a GPI-anchored protein, is known to localize to the luminal surface of brain endothelial cells throughout the cortex and cerebellum where it enzymatically modulates carbon dioxide–bicarbonate balance (*74*–*76*). While no specific role in BBB crossing has previously been attributed to CA-IV, it was recently found to be among the mouse proteins most strongly positively correlated with plasma-protein uptake in the brain (slightly stronger than the often-targeted transferrin receptor) (*77*). This property was hypothesized at the time to be potentially useful for identifying receptors for enhanced BBB crossing (*78*). CA-IV is also expressed in the gastrointestinal tract, kidney, and lung (*79*, *80*), as well as taste receptor cells, where it allows us to sense carbonation (*81*). While LY6A expression in the kidney, heart, and liver (*82*) does not result in increased transduction there by PHP.B (*17*), detailed peripheral characterization of 9P31, 9P36, and other CA-IV–interacting AAVs will be required to determine potentially enhanced infectivity in CA-IV–expressing peripheral tissue.

CA-IV is broadly conserved across vertebrates and has similar CNS expression profiles in humans (*51*–*53*), with recent single-cell analyses of human brain vasculature confirming *CA4*’s expression in the human BBB (*83*, *84*). Thus, CA-IV–interacting AAVs are attractive candidates for translation across diverse model organisms and potentially in human gene therapies. Of note, however, is that both 9P31 and 9P36 AAVs display enhanced potency with mouse CA-IV but not rhesus macaque or human CA-IV. While neither virus would be expected to translate from mice to these species, we have identified a therapeutic target and mechanism for BBB crossing that may. The possibility for some specific engineered AAV–binding epitopes to experience genetic drift between even closely related species confronts all products of directed evolution whose intended final use differs from their selection conditions. This takes on increasing importance when considering the risk for failed trials to also preclude patients from future AAV treatments by eliciting cross-reactive neutralizing antibodies (*85*–*87*). Future rational engineering of AAVs against species-appropriate CA-IV, aided by our APPRAISE-AAV method, is a promising avenue for the generation of noninvasive vectors with enhanced CNS potency. Targeting CA-IV may also find application across diverse protein and chemical modalities (*63*).

In summary, using an accessible cell culture screen, we were able to identify diverse targets for enhanced BBB crossing by engineered AAV capsids. These include a receptor for potent research tools in mice, LY6C1, against which we evolved an enhanced capsid, AAV-PHP.eC, and a previously unknown target that is broadly conserved across species, CA-IV, against which future engineered capsids may be designed for confident translation across species, including in humans. Cautioned by the differential performance of early engineered AAVs evolved in mice in hosts of different genetic backgrounds (with potentially different BBB compositions), the gene therapy field has increasingly transitioned to directed evolution in NHPs to develop AAVs and other biologics. In addition to being significantly slower and more resource intensive, the 25 to 30 million years of evolutionary divergence between macaques and humans (*88*, *89*) may still prove a formidable barrier to human therapeutic translation, resulting in NHP capsids that might still fail in humans. Our studies highlight the importance of understanding engineered AAV mechanisms and demonstrate that high-throughput selections in rodents continue to play an important role in therapeutic target identification. By finding cognate receptors for mouse-selected AAVs via mechanism-agnostic directed evolution, we identified CA-IV as a previously unknown target for enhanced BBB receptor–mediated transcytosis across species, including humans, that next could be leveraged for both experimental and therapeutic access to the brain from the bloodstream.

## MATERIALS AND METHODS

### Plasmids

AAV capsid variants were subcloned into the pUCmini-iCAP-PHP.B backbone (Addgene ID: 103002). Single-stranded AAV (ssAAV) genomes used were pAAV:CAG-mNeonGreen (Addgene ID: 99134) and pAAV:CAG-2×NLS-EGFP [equivalent version with one nuclear localization signal (NLS), Addgene ID: 104061], as noted in figures and legends. Receptor candidate plasmids were purchased from GenScript. All selected open reading frame clones were introduced to a pcDNA3.1^+^/C-(K)-DYK backbone, except for *Lynx1* in pcDNA3.1, which was generously shared by J. Miwa (Lehigh). AAV capsid libraries were amplified from pCRII-9Cap-XE plasmid and subcloned into recombinant AAV (rAAV)-ΔCap-in-cis-Lox2 plasmid for transfection with AAV2/9 REP-AAP-ΔCap [library plasmids available upon request from the California Institute of Technology (Caltech) CLOVER Center] (*22*).

### Animals

All animal procedures were approved by the Caltech Animal Care and Use Committee and comply with all relevant ethical regulations. C57BL/6J (000664), BALB/cJ (000651), CBA/J (000656), nonobese diabetic/ShiLtJ (001976), Syn1-Cre (3966), GFAP-Cre (012886), Tek-Cre (8863), and Olig2-Cre (025567) mouse lines were purchased from JAX. Heterozygous *Car4* KO mice (008217) were cryo-recovered by JAX and bred at Caltech to generate homozygous WT/WT and KO/KO animals. Six- to 8-week-old mice were intravenously injected with rAAV into the retro-orbital sinus. Mice were randomly assigned to a particular rAAV during testing of transduction phenotypes. Experimenters were not blinded for any of the experiments performed in this study.

### AAV vector production

AAV packaging and purification were performed as previously described (*31*). Briefly, rAAV was produced by triple transfection of HEK293T cells (American Type Culture Collection, CRL-3216) using polyethylenimine. Medium was collected at 72 and 120 hours after transfection, and virus was precipitated in 40% polyethylene glycol in 2.5 M NaCl. This was resuspended and combined with cell pellets 120 hours after transfection at 37°C in 500 mM NaCl, 40 mM tris, 10 mM MgCl_2_, and salt-active nuclease (100 U ml^−1^; ArcticZymes, 70910-202). The resulting lysate was extracted from an iodixanol (Cosmo Bio USA, OptiPrep, AXS-1114542) step gradient following ultracentrifugation. Purified virus was concentrated and buffer-exchanged with phosphate-buffered saline (PBS) before titer determination by quantitative polymerase chain reaction (PCR).

### AAV vector administration, tissue processing, and imaging

AAV vectors were administered intravenously to adult mice via retro-orbital injection at doses of 1 × 10^11^ or 3 × 10^11^ v.g. as indicated in figures and legends. After 3 weeks of expression, mice were anesthetized with Euthasol (pentobarbital sodium and phenytoin sodium solution, Virbac AH) and transcardially perfused with roughly 50 ml of 0.1 M PBS (pH 7.4) and then another 50 ml of 4% paraformaldehyde (PFA) in 0.1 M PBS. Organs were then harvested and postfixed in 4% PFA overnight at 4°C before being washed and stored in 0.1 M PBS and 0.05% sodium azide at 4°C. Last, the brain was cut into 100-μm sections on a Leica VT1200 vibratome. Images were acquired with a Zeiss LSM 880 confocal microscope using a Plan-Apochromat 10× 0.45 M27 (working distance, 2.0 mm) objective and processed in ZEN Black 2.3 SP1 (Zeiss) and ImageJ software.

### Single-cell RNA sequencing analyses

Analyses were performed on a preexisting C57BL/6J cortex single-cell RNA sequencing dataset with custom-written scripts in Python 3.7.4 using a custom fork off of scVI v0.8.1 and Scanpy v1.6.0, as described previously (*48*). Briefly, droplets that passed quality control were classified as “neurons” or “nonneurons” using a trained scANVI cell type classifier, retaining only those cells above a false discovery rate threshold of 0.05 after correction for multiple comparisons. Nonneuronal cells were further subtyped using a trained scVI model and clustered on the basis of the learned latent space using the Leiden algorithm as implemented in Scanpy. Endothelial cell clusters were assigned if they were positive for all marker genes for that cell subtype. Membrane proteins were filtered by UniProt keyword “cell membrane,” and differential expression scores were calculated in Scanpy.

### Immunofluorescence

Immunofluorescence experiments were performed on HEK293T cells to label transiently transfected receptors such as LY6A (1:200 dilution; Abcam, ab51317), LY6C1 (1:200 dilution; Abcam, ab15627), and CA-IV (1:40 dilution; Invitrogen, PA5-47312). HEK293T cells were seeded at 80% confluency in six-well plates and maintained in Dulbecco’s modified Eagle’s medium (DMEM) supplemented with 5% fetal bovine serum (FBS), 1% nonessential amino acids (NEAAs), and penicillin-streptomycin (100 U/ml) at 37°C in 5% CO_2_. Membrane-associated receptor candidates were transfected by polyethylenimine (PolySciences, no. 23966). Cells were seeded on Neuvitro poly-d-lysine–coated sterile German glass coverslips (Fisher Scientific, no. NC0343705) 24 hours after transfection in 24-well plates then fixed in 4% PFA once attached. Coverslips were blocked with 1× tris-buffered saline (TBS) containing 3% bovine serum albumin (BSA) for 30 min and incubated in primary antibody in 1× TBS, 3% BSA, and 0.05% Triton X-100 for 60 min at ambient temperature. Coverslips were washed three times in 1× TBS and then incubated with secondary antibody (LY6A and LY6C1, 1:1000 dilution; Invitrogen, A-21247; CA-IV, 1:1000 dilution; Invitrogen, A-21432) in the same medium for 60 min. Coverslips were mounted on slides with Diamond antifade mounting media with 4′,6-diamidino-2-phenylindole (Invitrogen, P36931). Fluorescent microscopic images were captured on a confocal laser scanning microscope (LSM 880, Carl Zeiss, USA).

### Cell culture characterization of rAAV vectors

HEK293T cells were seeded at 80% confluency in six-well plates and maintained in DMEM supplemented with 5% FBS, 1% NEAA, and penicillin-streptomycin (100 U ml^−1^) at 37°C in 5% CO_2_. Membrane-associated receptor candidates were transiently expressed in HEK293T cells by transfecting each well with 2.53 μg of plasmid DNA. Receptor-expressing cells were transferred to 96-well plates at 20% confluency and maintained in FluoroBrite DMEM supplemented with 0.5% FBS, 1% NEAA, penicillin-streptomycin (100 U ml^−1^), 1× GlutaMAX, and 15 mM Hepes at 37°C in 5% CO_2_. Cells expressing each receptor candidate were transduced with engineered AAV variants at 1 × 10^9^ and 5 × 10^8^ v.g. per well in triplicate. Plates were imaged 24 hours after transduction with the Keyence BZ-X700 using the 4× objective and NucBlue LiveReadyProbes reagent (Hoechst 33342) to autofocus each well.

### Cell culture fluorescence image quantitation

All image processing was performed using our custom Python image processing pipeline, available at github.com/GradinaruLab/in-vitro-transduction-assay. Briefly, the area of cells is determined in both bright-field and signal images, and the percent of cells transduced and brightness per transduced area is determined from these images.

First, background subtraction is performed on the bright-field images by applying Gaussian blur (skimage.filters.gaussian; sigma, 30; truncate, 0.35) and subtracting the product from the original bright-field image. In bright-field images, cells are silhouetted by the lamp producing both bright and dark edges. Histogram-based thresholding can be applied to these images to determine bright and dark regions of the bright-field image, which can be combined to create a mask of cell edges in the image. Cells can be filled by applying skimage.morphology.closing, which runs a template over the image to fill contiguous regions (skimage.morphology.disk; radius, 2). The total area of cells in the bright-field image can then be determined by summing all the pixels in the mask.

On the signal images, background subtraction is performed by applying Gaussian blur (skimage.filters.gaussian; sigma, 100). Subtracting the product of Gaussian blur from the original signal image produces an image with minimal fluctuations in background intensity. Histogram-based thresholding is applied to this image to identify the intensity of background in the bright-field image and create a mask of bright regions in the image, which is composed of transduced cells. Noise can be removed from the mask using skimage.morphology.remove_small_objects (min_size, 5). From this, the total area of transduced cells can be determined by summing up all the pixels in the mask.

After performing this segmentation, the percentage of cells transduced can be determined by taking the ratio of signal area to the total cell area. By multiplying the mask by the original image and summing up all the pixel intensities in the product image, the total brightness of transduced cells can be determined. This value can then be divided by the total area of transduced cells to determine the brightness per transduced area.

### Receptor protein production

LY6A-Fc was produced in Expi293F suspension cells grown in Expi293 expression medium (Thermo Fisher Scientific) in a 37°C and 5% CO_2_ incubator with shaking at 130 rpm. Transfection was performed with Expifectamine according to the manufacturer’s instructions (Thermo Fisher Scientific). Following harvesting of cell-conditioned media, 1 M tris (pH 8.0) was added to a final concentration of 20 mM. Ni–nitrilotriacetic acid (NTA) agarose (QIAGEN) was added to ∼5% conditioned media volume. One-molar sterile PBS (pH 7.2; GIBCO) was added to ∼3× conditioned media volume. The mixture was stirred overnight at 4°C. Ni-NTA agarose beads were collected in a Buchner funnel and washed with ∼300 ml of protein wash buffer [30 mM Hepes (pH 7.2), 150 mM NaCl, and 20 mM imidazole]. Beads were transferred to an Econo-Pac chromatography column (Bio-Rad), and protein was eluted in 15 ml of elution buffer [30 mM Hepes (pH 7.2), 150 mM NaCl, and 200 mM imidazole]. Proteins were concentrated using Amicon Ultracel 10K filters (Millipore), and absorbance at 280 nm was measured using a NanoDrop 2000 spectrophotometer (Thermo Fisher Scientific) to determine protein concentration.

### Surface plasmon resonance

SPR was performed using a Sierra SPR-32 (Bruker). LY6A-Fc fusion protein in HBS-P^+^ buffer (GE Healthcare) was immobilized to a protein A sensor chip at a capture level of approximately 1200 to 1500 response units. Twofold dilutions of rAAVs beginning at 2 × 10^12^ v.g. ml^−1^ were injected at a flow rate of 10 μl min^−1^ with a contact time of 240 s and a dissociation time of 600 s. After each cycle, the protein A sensor chip was regenerated with 10 mM glycine (pH 1.5). Kinetic data were double reference–subtracted.

### Automated pairwise peptide receptor analysis for screening engineered AAVs

Details of the APPRAISE-AAV method are described in Ding *et al.* (*45*). APPRAISE can be accessed through a web-based Jupyter notebook (https://tiny.cc/APPRAISE), and a local version of the software is available on GitHub (https://github.com/GradinaruLab/APPRAISE). Briefly, FASTA format files containing a target receptor amino acid sequence (mature protein part only) and peptide sequences corresponding to amino acids 587 to 594 (WT AAV9 VP1 indices) from two AAV capsids of interest were used for structural prediction using a batch version of ColabFold (*90*) (AlphaFold-ColabFold version 2.1.14), a cloud-based implementation of multiple sequence alignment (*91*–*93*), and AlphaFold-Multimer (*47*). The ColabFold Jupyter notebook was run on a Google Colaboratory session using a graphics processing unit (GPU; NVIDIA Tesla V100 SXM2 16 gigabytes; we found that the same model of the GPU yielded the most consistent results). We chose alphafold-multimer-v2 as the default AlphaFold version unless otherwise specified. Each model was recycled three times, and 10 models were generated from each competition. Models were quantified with PyMol (version 2.3.3) using a custom script to count the total number of atoms in the interface ($N_{\mathrm{contact}}^{\mathrm{POI}}$, defined by a distance cutoff of 5 Å), the total number of atoms in the peptide that are clashing with the receptor ($N_{\mathrm{clash}}^{\mathrm{POI}}$, defined by a distance cutoff of 1 Å), the binding angle of the peptide (θ, defined as the angle between the vector from receptor gravity center to receptor anchor and the vector from receptor gravity center to peptide gravity center), and the binding depth (*d*, defined as the difference of the distance between the closest point on the peptide to the receptor center and the minor radius of the ellipsoid hull of the receptor normalized by the minor radius) of the peptide in each putative peptide-receptor complex model. The minor radii of the ellipsoid hulls of receptors were measured using HullRad 8.1 89 (LY6A, 13.4 Å; LY6C1, 12.7 Å; mouse CA-IV, 23.0 Å). Last, the metric Δ*B*^POI,competitor^ for ranking the propensity of receptor binding was calculated by subtracting the total binding score of the competing peptide from the corresponding score for the peptide of interest

$$\begin{array}{c} {\text{Δ}B}^{\mathrm{POI},\mathrm{competitor}}=B^{\mathrm{POI}}-B^{\mathrm{competitor}}\\ =\max(B_{\mathrm{energetic}}^{\mathrm{POI}}+B_{\mathrm{angle}}^{\mathrm{POI}}+B_{\mathrm{depth}}^{\mathrm{POI}},0)\\ \;-\max(B_{\mathrm{energetic}}^{\mathrm{competitor}}+B_{\mathrm{angle}}^{\mathrm{competitor}}+B_{\mathrm{depth}}^{\mathrm{competitor}},0) \end{array}$$

where the individual terms are defined as follows

$$B_{\mathrm{energetic}}^{\mathrm{peptide}}=\max(N_{\mathrm{contact}}^{\mathrm{peptide}}-10^{3}\cdot N_{\mathrm{clash}}^{\mathrm{peptide}},0)$$

$$B_{\mathrm{energetic}}^{\mathrm{peptide}}=\max(N_{\mathrm{contact}}^{\mathrm{peptide}}-10^{3}\cdot N_{\mathrm{clash}}^{\mathrm{peptide}},0)$$

$$B_{\mathrm{depth}}^{\mathrm{peptide}}=10^{2}\cdot d^{3}$$

The mean number of this metric across replicates was used to form a matrix and plot a heatmap. Peptides in the heatmap were ranked by the total number of competitions each peptide won minus the total number of competitions it lost (competitions with Δ*B*^POI,competitor^). Scores that have *P* values greater than 0.05 in the one-sample Student’s *t* test were excluded.

### Computational structure modeling of receptor-AAV complexes

Peptide-receptor structures were modeled using a similar procedure as described in the APPRAISE-AAV section but with only one single peptide of interest in the input file to achieve higher accuracy. AAV trimer receptor complex models were produced using an integrative structure modeling method (fig. S6A). Trimers at the AAV threefold symmetry interface were chosen as the minimal complete binding interface with a putative receptor that might recapitulate the entire viral particle while optimizing computational efficiency. First, a peptide-receptor model was generated by modeling the 15-amino-acid peptide sequence between the residues 587 and 594 (both in WT AAV9 VP1 indices) from the AAV variant of interest in complex with the target receptor as described above. Then, a trimer model of the AAV variant of interest was modeled using AlphaFold-Multimer. The two residues with the highest confidence score (pLDDT score) in the 15-amino-acid peptide of the peptide-receptor model, Pro5′ and Phe6′, were structurally aligned to the corresponding residues on the first chain of the trimer model. A coarse combined model was then generated by combining the receptor and the two high-confidence AAV residues from the peptide receptor model with the remaining AAV residues from the trimer model. The two loops between Pro5′ and Phe6′ and the high-confidence AAV9 backbone in the coarse combined model [corresponding to residues 588-(588+)4′ and residues (588+)7′-590, respectively] were then individually remodeled using RosettaRemodel (*59*) from the Rosetta software bundle (release 2018.48.60516). Last, these remodeled loops were merged to generate a final model. The pLDDT scores for individual residues from the original AlphaFold-Multimer outputs were used to color images of the final model.

### M-CREATE selections for PHP.eC

AAV capsid libraries were produced, administered, and recovered after selection in vivo for next-generation sequencing (NGS), as described previously (*22*). Briefly, an initial library was generated by pooling three-amino-acid NNK substitutions that scan from AAV9 587 through the 7-amino-acid insertion to AAV9 position 590 of AAV-PHP.C1, AAV-PHP.C2, AAV-PHP.C3, and AAV-PHP.C4 (Fig. 4A). A custom-designed synthetic round 2 library containing degenerate codon duplicates of 5515 capsid variants identified from the round 1 library post-selection and spike-in controls was synthesized in an equimolar ratio by Twist Biosciences. To prevent capsid mosaicism, only 10 ng of assembled library was transfected per 150-mm dish of 293 T producer cells, and assembled capsids were purified at 60 hours after transfection. Round 1 library was retro-orbitally injected at 5 × 10^10^ v.g. per mouse in Syn-Cre mice, while round 2 library was retro-orbitally injected at 5 × 10^11^ v.g. per mouse in Syn-Cre, GFAP-Cre, Tek-Cre, Olig2-Cre, WT C57BL/6J, and WT BALB/cJ mice.

Two weeks after injection, mice were euthanized, and all organs including the brain were collected, snap-frozen on dry ice, and stored at −80°C. Frozen tissue was homogenized using BeadBug homogenizers (Homogenizers, Benchmark Scientific, D1032-15, D1032-30, and D1033-28), and rAAV genomes were TRIzol-extracted. Purified rAAV DNA (Zymo DNA Clean and Concentrator Kit, D4033) was amplified by PCR using Cre-dependent primers, adding flow cell adaptors around the diversified region for NGS. NGS data were aligned and processed as described previously to extract round 1 variant sequence counts and round 2 variant enrichment scores.

### Immunohistochemistry

Immunohistochemistry was performed on 100-μm-thick tissue sections incubated in blocking buffer [10% normal donkey serum, 0.1% Triton X-100, and 0.01% sodium azide in 0.1 M PBS (pH 7.4)] with cell-type markers including NeuN (1:400; Abcam, ab177487; 1:400; Sigma-Aldrich, ABN91) for neurons, S100 (1:400; Abcam, ab868) for astrocytes, and Olig2 (1:400; Abcam, ab109186) for oligodendrocyte lineage cells for 24 hours at room temperature with rocking. Tissues were then washed one to three times with wash buffer [0.1% Triton X-100 in 0.1 M PBS (pH 7.4)] over a period of 5 to 6 hours. Incubation with secondary antibodies at the specified dilutions was done for a further 12 to 24 hours (1:500; anti-rabbit Alexa Fluor 568, Abcam, ab175470; 1:500; anti-chicken Alexa Fluor 647, Abcam, ab150171) and then further washed three times. Stained tissue sections were mounted with ProLong Diamond antifade mountant (Thermo Fisher Scientific, P36970).
