# Supplementary material for: Primate-conserved carbonic anhydrase IV and murine-restricted LY6C1 enable blood-brain barrier crossing by engineered viral vectors
Source: Sci Adv. 2023 Apr 19;9(16):eadg6618. doi: 10.1126/sciadv.adg6618 (PMC10115422; doi:10.1126/sciadv.adg6618)
Supplement: Supplementary file 1 — Table S1 Figs. S1 to S6 [file sciadv.adg6618_sm.pdf]

Science Advances
AAAS

Supplementary Materials for

Primate-conserved carbonic anhydrase IV and murine-restricted LY6C1 enable blood-brain barrier crossing by engineered viral vectors

Timothy F. Shay *et al.*

Corresponding author: Timothy F. Shay, tshay@caltech.edu; Viviana Gradinaru, viviana@caltech.edu

Sci. Adv. **9**, eadg6618 (2023)
DOI: 10.1126/sciadv.adg6618

This PDF file includes:

Table S1
Figs. S1 to S6

Name	Accession No.	Mean Expression	Differential Expression
Abcb1a	NM_011076.2	0.000688904	7.94237100
Abcg2	NM_011920.3	0.000745241	5.56071470
Acvrl1	NM_009612.3	0.000485079	6.78613330
Adgrf5	NM_001357332.1	0.000546496	7.45316400
Adgrl4	NM_133222.3	0.000763887	8.24139400
App	NM_001198823.1	0.000845707	0.04600084
Bsg	NM_009768.2	0.013027801	4.68415070
Car4	NM_007607.2	0.000713242	3.71078540
Cd34	NM_133654.3	0.000467251	3.09839130
Cdh5	NM_009868.4	0.000332645	7.48680500
Cldn5	NM_013805.4	0.005443705	9.17483200
Clec2d	NM_053109.3	0.000584914	6.37040600
Clic1	NM_033444.2	0.000377934	2.64024160
Clic4	NM_013885.2	0.000408817	3.14403030
Eng	NM_007932.2	0.000646813	5.99654530
Esam	NM_027102.3	0.000660755	6.04634760
Fcgrt	NM_010189.3	0.000503876	4.02808500
Flt1	NM_010228.3	0.002267079	8.53988600
Ifitm2	NM_030694.1	0.000314049	3.51603910
Ifitm3	NM_025378.2	0.001015794	5.31335400
Igf1r	NM_010513.2	0.000750016	3.37413700
Itm2b	NM_008410.2	0.001596154	-0.10450481
Kdr	NM_010612.2	0.000304256	6.83441160
Kitl	NM_013598.3	0.000382663	3.28041740
Ly6a	NM_010738.3	0.002695857	8.70076600
Ly6c1	NM_010741.3	0.003812270	8.84188900
Ly6e	NM_008529.3	0.001050145	2.95476170
Ocln	NM_008756.2	0.000208863	6.83609100
Paqr5	NM_028748.2	0.000262998	7.77428870
Pecam1	NM_008816.3	0.000423881	7.13435360
Podxl	NM_013723.3	0.000407256	6.36354730
Prom1	NM_001163577.1	0.000271125	5.45687630
Serinc3	NM_012032.4	0.000562215	1.23178270
Slc22a8	NM_001164634.1	0.000243451	5.05416900
Slc2a1	NM_011400.3	0.001843255	5.43814370
Slc6a6	NM_009320.4	0.001148025	3.84664630
Slc7a1	NM_007513.4	0.000403344	4.17060850
Slco1A4	NM_030687.1	0.001701704	8.39613200
Slco1c1	NM_021471.2	0.000828427	3.68831500
Tek	NM_013690.3	0.000224058	7.49632500

Table S1: Single-cell RNA sequencing profiles of the C57BL/6J brain endothelial cell membrane proteins included in cell culture screen.

Supplementary Figures

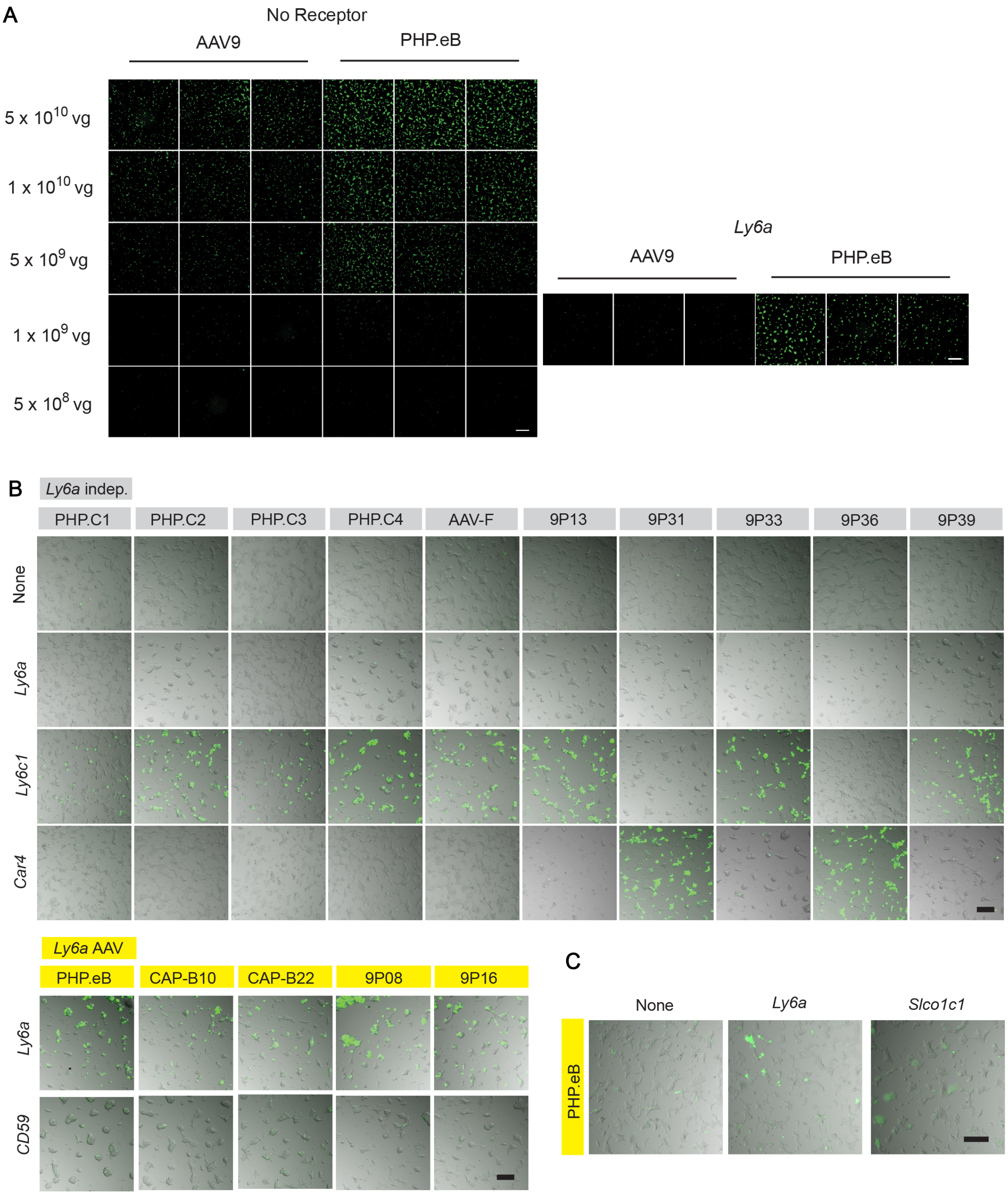

Fig. S1. Representative images from cell culture infectivity assay. A, Fluorescence images showing the dose dependence of AAV9 and PHP.eB packaging CAG-mNeonGreen in HEK293T cells in 96-well plates. At 1 x 10^9 v.g. per well, PHP.eB has markedly higher potency than AAV9 in *Ly6a*-transfected HEK293T cells. Representative images are shown. Scale bars = 500 µm. **B,** Representative images of HEK cells cultured in 96-well plates, either mock-transfected (none) or transfected with a potential receptor, infected with 1 x 10^9 v.g. per well of PHP.B sequence family AAVs (yellow label background indicating LY6A binding) or *Ly6a*-independent AAVs (gray label background) packaging CAG-mNeonGreen and imaged 24 hours post transduction. An overlay of brightfield and fluorescence images is presented. Scale bars = 200 µm. **C,** Representative images of HEK cells cultured in 96-well plates, either mock-transfected (none) or transfected with *Ly6a* or *Slco1c1*. *Slco1c1*-transfected cells display dim and diffuse fluorescence that extends beyond cell boundaries when infected with every AAV tested. An overlay of brightfield and fluorescence images is presented. Scale bar = 200 µm.

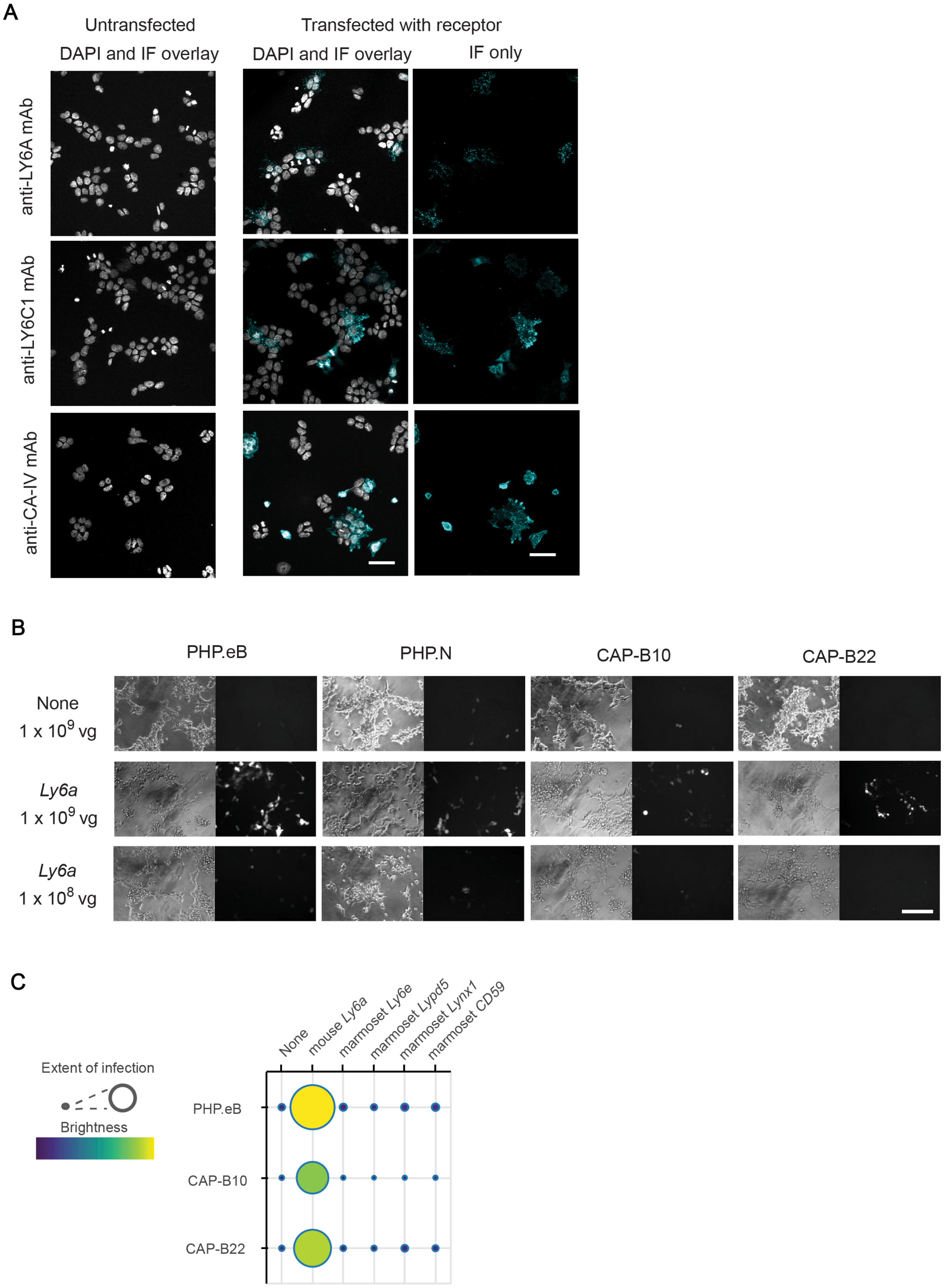

A
Untransfected
DAPI and IF overlay
Transfected with receptor
DAPI and IF overlay
IF only
anti-LY6A mAb
anti-LY6C1 mAb
anti-CA-IV mAb
B
PHP.eB
PHP.N
CAP-B10
CAP-B22
None
1 x 10^9 vg
Ly6a
1 x 10^9 vg
Ly6a
1 x 10^8 vg
C
Extent of infection
Brightness
None
mouse Ly6a
marmoset Ly6e
marmoset Lypd5
marmoset Lynx1
marmoset CD59
PHP.eB
CAP-B10
CAP-B22

Fig. S2. Immunofluorescence confirms expression of selected membrane proteins from the cell culture screen. A, DAPI and immunofluorescence (IF) overlay for untransfected and receptor-transfected HEK293T cells stained with the indicated antibody as well as the IF channel alone from receptor-transfected HEK293T cell images (at left). Scale bars = 50 μm. **B,** LY6A-interacting AAVs engineered from PHP.eB display the same degree of potency enhancement in cell culture. Brightfield (*left*) and fluorescence (*right*) images of PHP.eB, PHP.N, and CAP-B10 packaging CAG-mNeonGreen as well as CAP-B22 packaging CAG-NLS-GFP at two different doses in mock-transfected and LY6A-expressing cells. Scale bar = 100 μm. **C**, CAP-B10 and CAP-B22 do not display a potency enhancement with marmoset Ly6 proteins. Potency of engineered AAVs for HEK293T cells transfected with the potential receptor panel. Extent of infection (Max: 0.52, Min: 0.05), Total brightness per signal area (Max: 0.79, Min: 0.17).

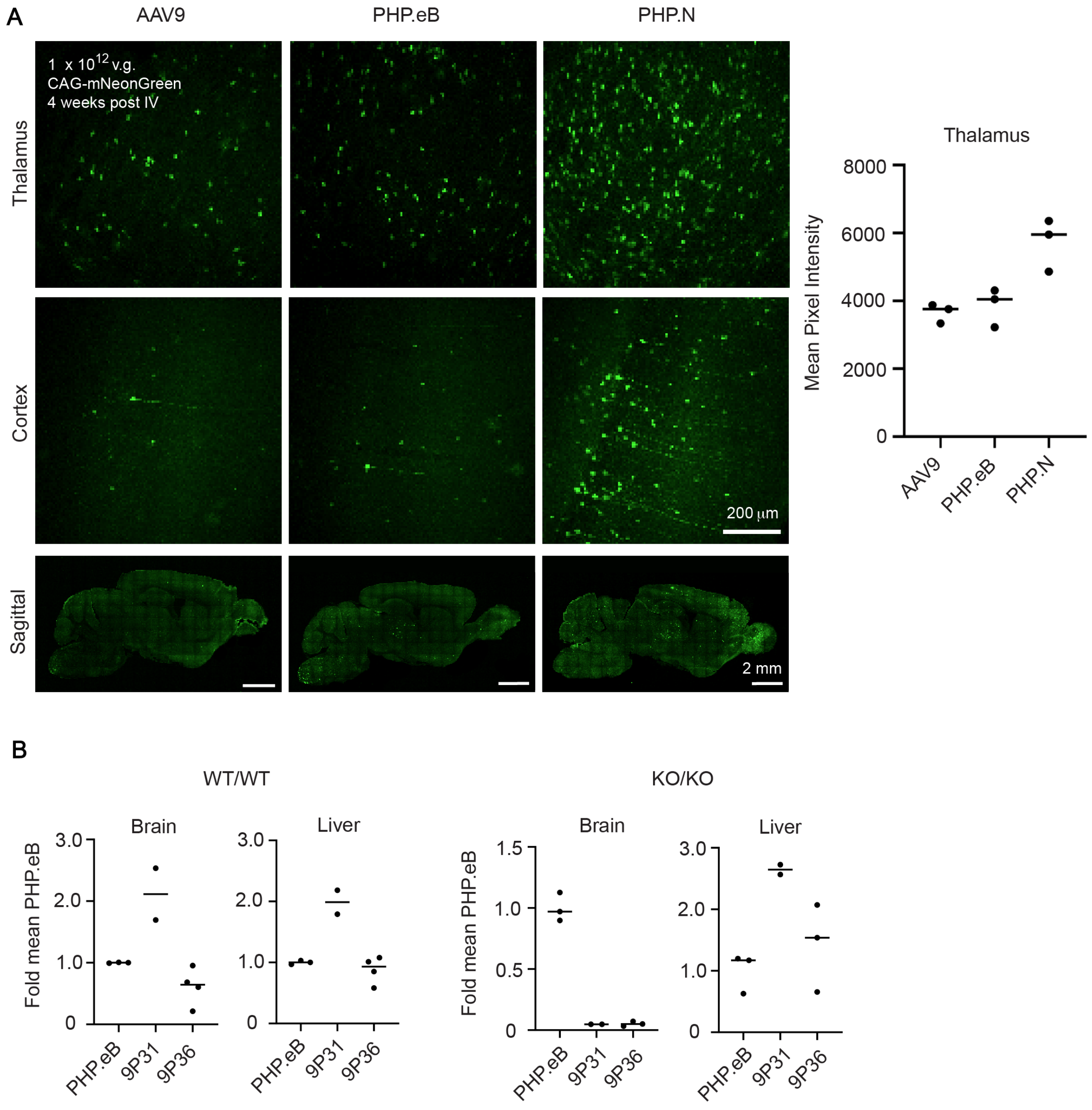

Fig. S3. In CBA/J mice at high doses, PHP.N outperforms AAV9 and PHP.eB. A, Animals were retro-orbitally injected at 6-8 weeks old with 1 x 10^{12} v.g. of AAV9, PHP.eB, or PHP.N packaging CAG-mNeonGreen (n = 3). Four weeks after injection, tissue was collected and imaged to detect virus potency. Each data point in the thalamus mean pixel intensity quantitation represents one animal and bars indicate the mean value. **B,** Quantification of AAV potency in WT and *Car4*-KO mice. Mean pixel intensity of whole brain sagittal slices and liver sections were quantified and

each individual datapoint was then normalized to the mean PHP.eB intensity. Each data point represents one animal and bars indicate the mean value.

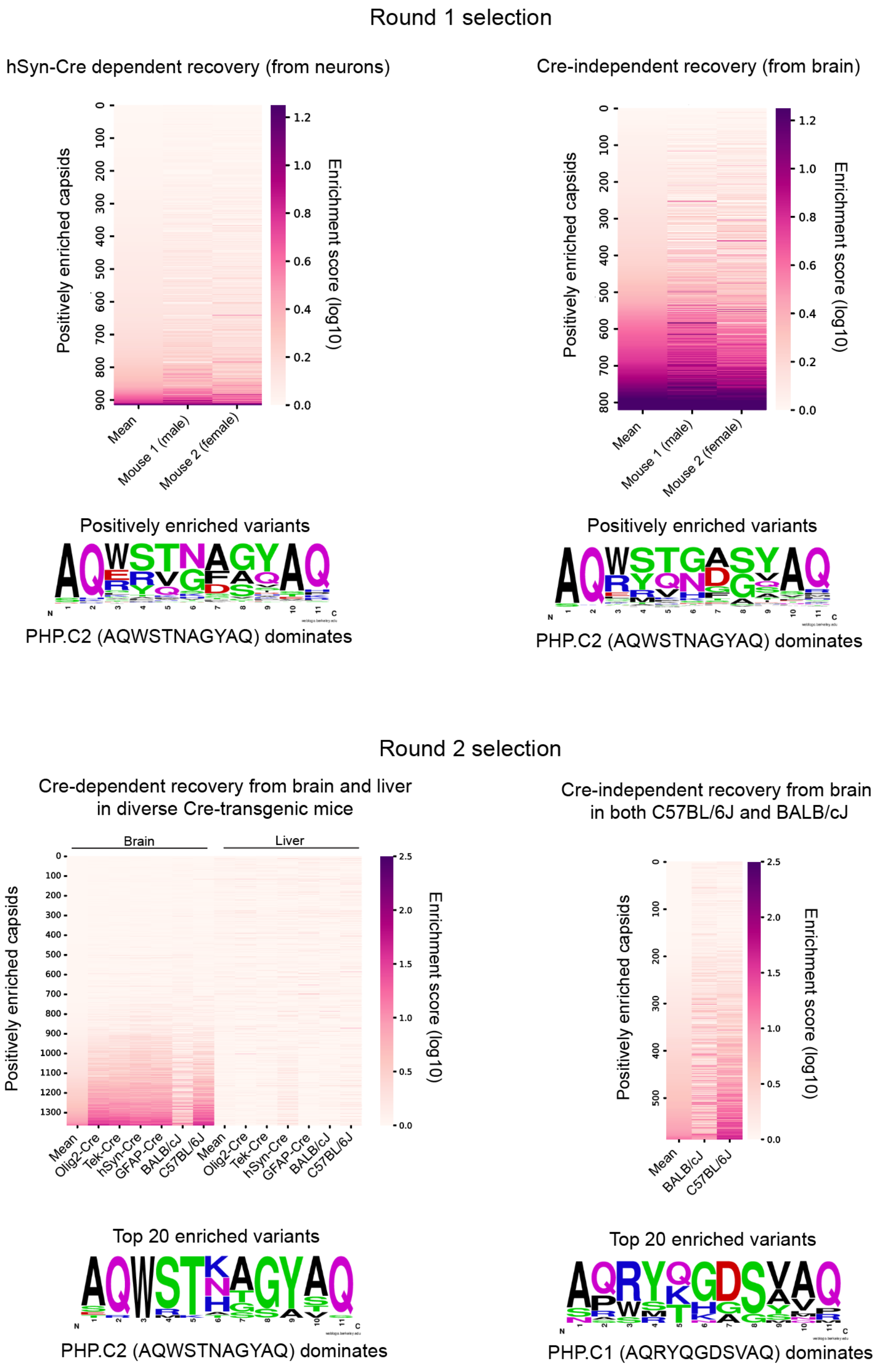

Fig. S4. Enriched capsid variants during M-CREATE selections. Capsids ranked according to their enrichment score from round 1 (top) and round 2 (bottom) selections using M-CREATE Cre-

dependent recovery (left) or Cre-independent recovery (right). Sequence frequency plots indicate the patterns of the most enriched variants from each round and selection type.

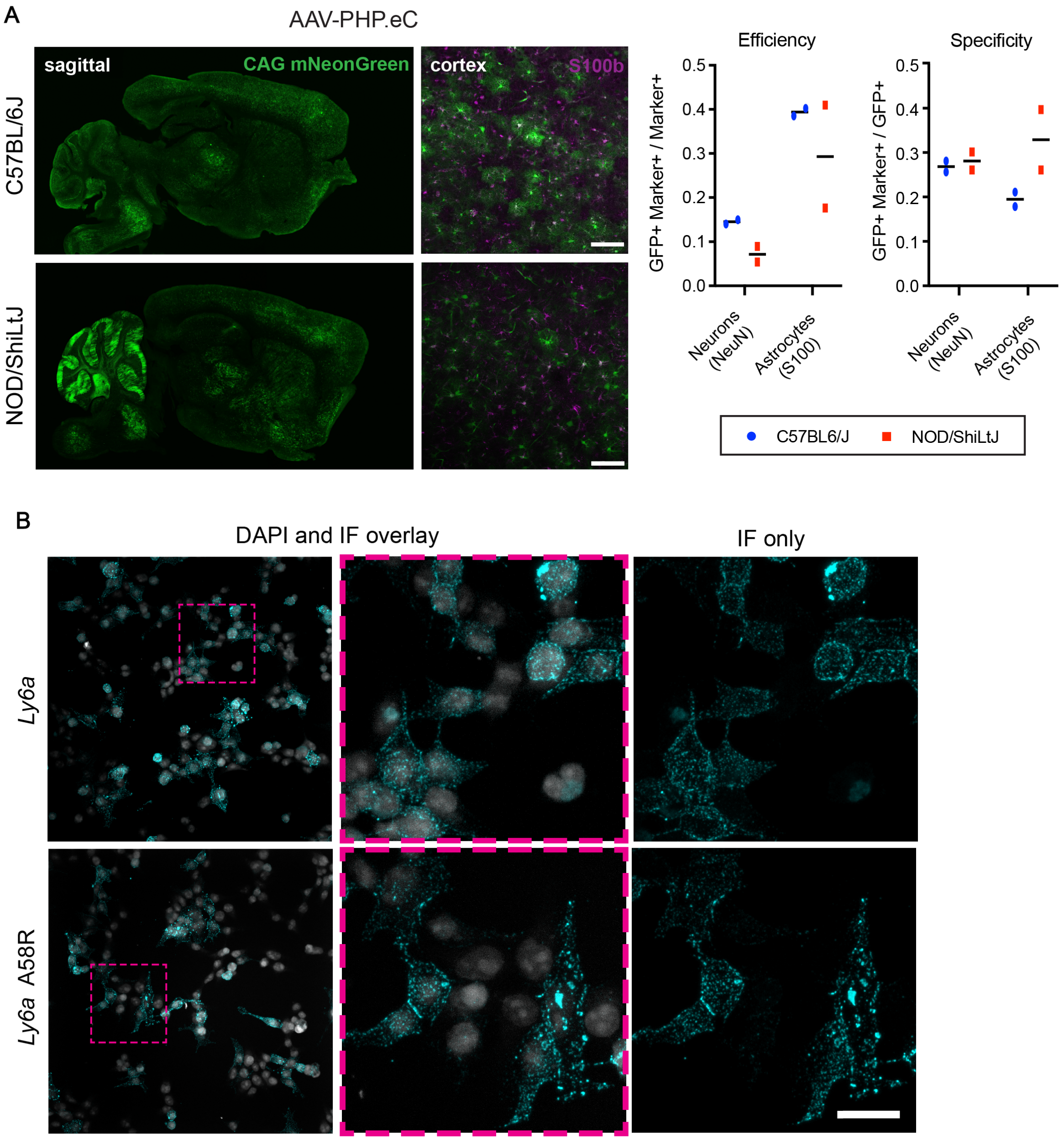

Fig. S5. Characterization of AAV-PHP.eC brain cell type tropism. A, Adult 6 to 8-week old C57BL/6J and NOD/ShiLtJ mice were retro-orbitally injected with 3 x 10^{11} v.g. each of AAV-PHP.eC packaging CAG mNeon Green. After 3 weeks, tissues were stained with the indicated cell-type markers and imaged to allow tropism quantification. Cortical slices were stained with NeuN or S100 for neurons and astrocytes, respectively. Scale bars = 100 µm. **B,** DAPI and

immunofluorescence (IF) of wild type and A58R LY6A transiently overexpressed in HEK293T cells, with zoomed center column and zoomed IF only in the right column. Scale bar = 20 μm.

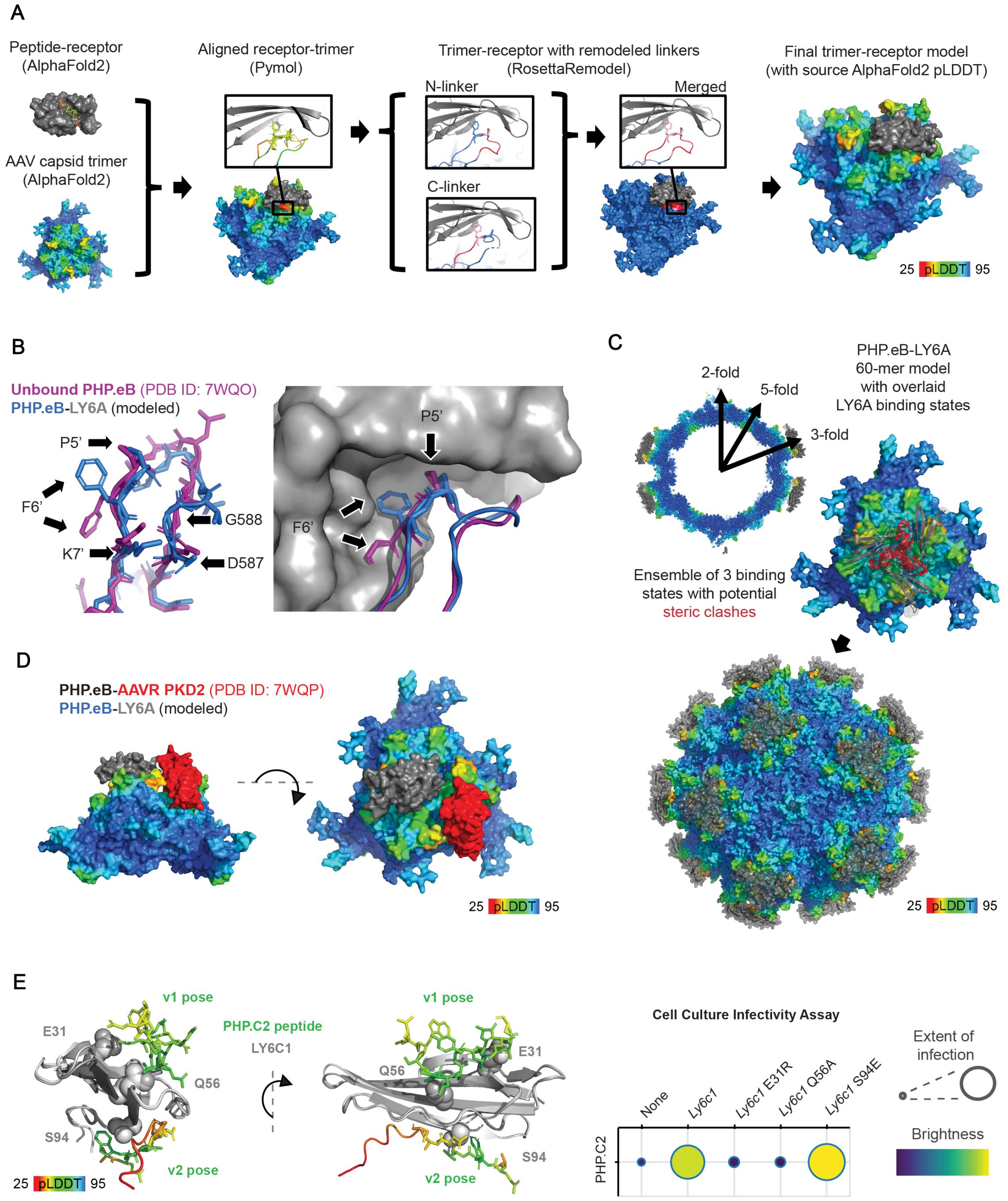

Fig. S6. An integrative structure modeling method yields a snapshot of PHP.eB-LY6A interaction. A, A workflow for modeling engineered AAV-receptor complex structures (PHP.eB: color-coded by pLDDT score, LY6A: grey). **B,** Comparison between computationally modeled PHP.eB-LY6A (PHP.eB: blue, LY6A: grey) and CryoEM structure of unbound PHP.eB (purple,

PDB ID: 7WQO). All high-confidence residues (pLDDT > 45, see left panel arrow) within the inserted peptide showed consistent conformations between the two models except for F6’, which did not have clear side chain density in the CryoEM map. The side chain of F6’ predicted in the unbound PHP.eB model would cause a steric clash in the predicted complex model. **C,** Assembled PHP.eB capsid-LY6A model representing an ensemble of all LY6A binding states, despite steric clashes, as would occur in cryo-EM particle reconstruction (PHP.eB: rainbow, LY6A:grey)**.** Top: cross section of the assembled capsid model. Middle: zoom-in of a 3-fold spike, highlighting steric clashes (red) between three different binding states. **D**, Overlaid structures of computationally modeled PHP.eB-LY6A (PHP.eB: rainbow, LY6A: grey) and CryoEM model of PHP.eB-AAVR PKD2 (AAVR PKD2: red, PDB ID: 7WQP). **E *left*,** Comparison between the computationally modeled PHP.C2-LY6C1 binding poses predicted by AlphaFold-Multimer v1 and v2. PHP.C2 peptides are color-coded by residue according to pLDDT score. LY6C1 residues chosen for mutation are shown as spheres. ***right,*** Potency of PHP.C2 for HEK293T cells transfected with wild type or mutant LY6C1 receptors. Extent of infection (Max: 0.67, Min: 0.13), Total brightness per signal area (Max: 0.72, Min: 0.31).
